# Case report: Rare oral manifestations in Cowden syndrome with *PTEN* mutation

**DOI:** 10.3389/fonc.2024.1323225

**Published:** 2024-02-09

**Authors:** Wei Yuan, Yanbin Liu, Haibin Sun, Ming Su, Lizheng Qin, Xin Huang

**Affiliations:** ^1^ Department of Oral and Maxillofacial-Head and Neck Oncology, Beijing Stomatological Hospital, Capital Medical University, Beijing, China; ^2^ Department of Dental Implant Center, Beijing Stomatological Hospital, Capital Medical University, Beijing, China

**Keywords:** Cowden syndrome, PTEN mutation, oral lesions, clinical manifestations, genetic testing

## Abstract

**Background:**

Cowden syndrome (CS) is a rare genetic disorder associated with *PTEN* gene mutations. It is characterized by macrocephaly, specific mucocutaneous features, and a predisposition to benign and malignant tumors. Cases of CS primarily presenting with oral clinical manifestations are relatively uncommon.

**Methods/Results:**

We report the case of a 41-year-old male proband who presented with bilateral commissural and lingual externally projecting symmetric lesions for over two years. The proband also exhibited other features, including macrocephaly, communication difficulties, and obesity. Similar oral clinical manifestations were observed in family members. Whole exome sequencing analysis revealed *PTEN* gene mutations associated with CS in both the proband and his younger brother. This case serves as a reminder to be aware of the diverse presentations of CS in oral clinical practice and highlights the importance of genetic testing for guiding diagnosis and treatment.

**Conclusion:**

There are few reported cases of CS primarily presenting with oral lesions. This finding contributes to further understanding of certain aspects of the pathogenesis of CS and enhances awareness of CS cases primarily exhibiting oral clinical manifestations.

## 1 Introduction

Cowden syndrome (CS; OMIM:158350) is a rare genetic disorder associated with *PTEN* (phosphatase and tensin homolog) gene mutations (1). CS exhibits highly diverse clinical features, typically including cutaneous manifestations such as multiple skin lesions, facial trichilemmomas, and acral keratosis. Additionally, CS patients may experience various visceral organ involvements, including thyroid abnormalities, breast cancer, endometrial cancer, renal cell carcinoma, and gastrointestinal polyps. Recent studies have also suggested an association between *PTEN* mutations and neurodevelopmental disorders, such as autism spectrum disorders and macrocephaly (2). CS was initially described by Lloyd and Dennis in 1963 and is now recognized as one of the *PTEN* hamartoma tumor syndromes (PHTS), along with other related conditions including Bannayan-Riley-Ruvalcaba syndrome and Proteus syndrome (3). CS follows an autosomal dominant inheritance pattern, meaning that an affected individual has a 50% chance of passing on the condition to each of their offspring (4, 5). Current research indicates that CS manifests diverse and distinctive features in oral clinical presentations. These lesions encompass mucosal papules, warts, and multiple papillomatosis within the oral cavity (6–8). A comprehensive understanding of the relationship between CS, associated with *PTEN* gene mutations, and its oral clinical manifestations holds significant importance for improving the diagnosis, treatment, and management of this condition.

We present a case report of a rare instance of CS with unique oral manifestations. Whole exome sequencing (WES) identified a *PTEN* gene mutation associated with this condition. This article emphasizes the significance of early diagnosis and recognition of uncommon oral presentations in CS. Appropriate management and genetic counseling are crucial for this hereditary disorder.

## 2 Case description

**Figure 1:**
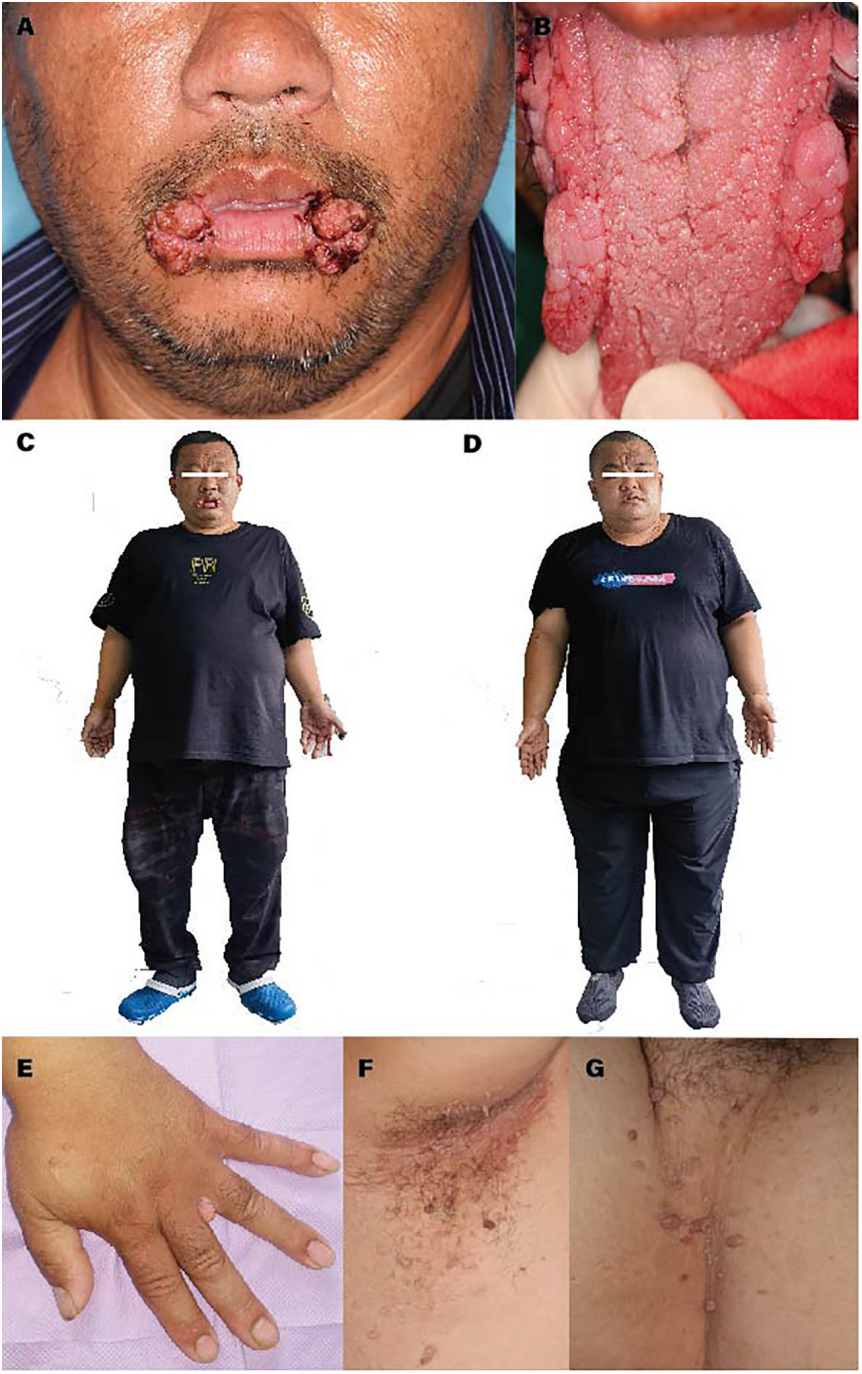
**(A)** Symmetric externally projecting lesions observed in the bilateral commissures of the mouth. **(B)** Symmetric externally projecting lesions located on both sides of the tongue, as well as scrotal tongue. **(C, D)** Physical characteristics observed in the proband and family members, including macrocephaly and overgrowth. **(E)** Lesions between the fingers on the palms. **(F)** Lesions in the axillary region. **(G)** Lesions in the inguinal region.

A 41-year-old male presented to the oral and maxillofacial surgery department with painless bilateral lesions in the angles of the mouth for over 2 years (**Figure 1A**). The proband reported initial onset on the right side followed by involvement of the left side, with subsequent appearance of similar lesions on both sides of the tongue after 6 months (**Figure 1B**). There were no associated symptoms such as pain, numbness, or restricted mouth opening. At the age of approximately 36, he had received cryotherapy treatment for bilateral angular lesions at an external dental clinic (diagnosis unknown). At the age of 40, the proband experienced a cerebral infarction and was concurrently diagnosed with hypertension, currently managed with medication. Family history investigation revealed that the proband’s younger brother had presented to our hospital at the age of 26 with bilateral angular lesions, which were subsequently excised using laser ablation. Pathological examination showed hyperplasia of stratified squamous epithelium with excessive surface keratinization, elongation and thinning of the epithelial rete ridges, indistinct basal membrane in some areas, lymphocyte infiltration in the lamina propria, plasma cell infiltration, and formation of micro abscesses. The proband’s father had been diagnosed with bilateral angular lesions at the age of 66 but remained untreated. He passed away 2 years later due to cardiovascular disease. The proband’s mother did not exhibit similar clinical symptoms and has since deceased. Clinical examination revealed speech and non-verbal communication impairments in the proband, along with macrocephaly in both the proband and his brother (head circumference: 66 cm; 69 cm), accompanied by excessive growth and obesity. **Figure 1C** illustrates the proband, while **Figure 1D** depicts the proband’s younger brother. Oral examination demonstrated externally projecting symmetric lesions in the angles of the mouth and lateral borders of the tongue, characterized by soft consistency, pedunculated appearance, and digitiform branching, along with fissured scrotal tongue. Skin examination revealed multiple firm, flesh-colored papillary lesions in the interdigital spaces of the palms, axillae, and groin regions (**Figures 1E, F**).

## 3 Results

After obtaining informed consent, blood samples were collected from the proband, the proband’s younger brother, and the proband’s cousin for WES.

WES primarily focuses on sequencing and detection of the entire exome portion of genes. The complete human genome consists of non-coding and coding regions, with the coding region further divided into exonic and intronic regions. The exome encompasses the entirety of the coding and contains crucial information required for protein synthesis. It encompasses a significant portion of functionally relevant variants associated with individual phenotypes. Thus, pathogenic variants within the exonic region are considered directly relevant to protein functional alterations, and therefore, WES is commonly employed in clinical settings to identify variant loci that assist in diagnosis. In study, sequencing was performed after the capture of the entire exome using exome probes. Due to the generation of extensive data through WES, including numerous variants that may be benign or common, a variant filtering strategy is necessary to identify disease-associated variants. The variant filtering strategy employed in this study was based on the following primary factors Firstly, filtering based on the basic data quality of mutation loci was conducted, where loci with sequencing coverage below 10x were excluded, with special consideration for further validation. Secondly, selection based on pathogenicity was performed according to the American College of Medical Genetics and Genomics (ACMG) guidelines, retaining pathogenic likely pathogenic/variants of uncertain significance while excluding variants classified as clearly benign/likely benign. Additionally, population frequency of the variants at each locus was considered, with variants below a frequency of 0.1% considered as low-frequency mutations, although different thresholds were employed for specific diseases.

**Figure 2:**
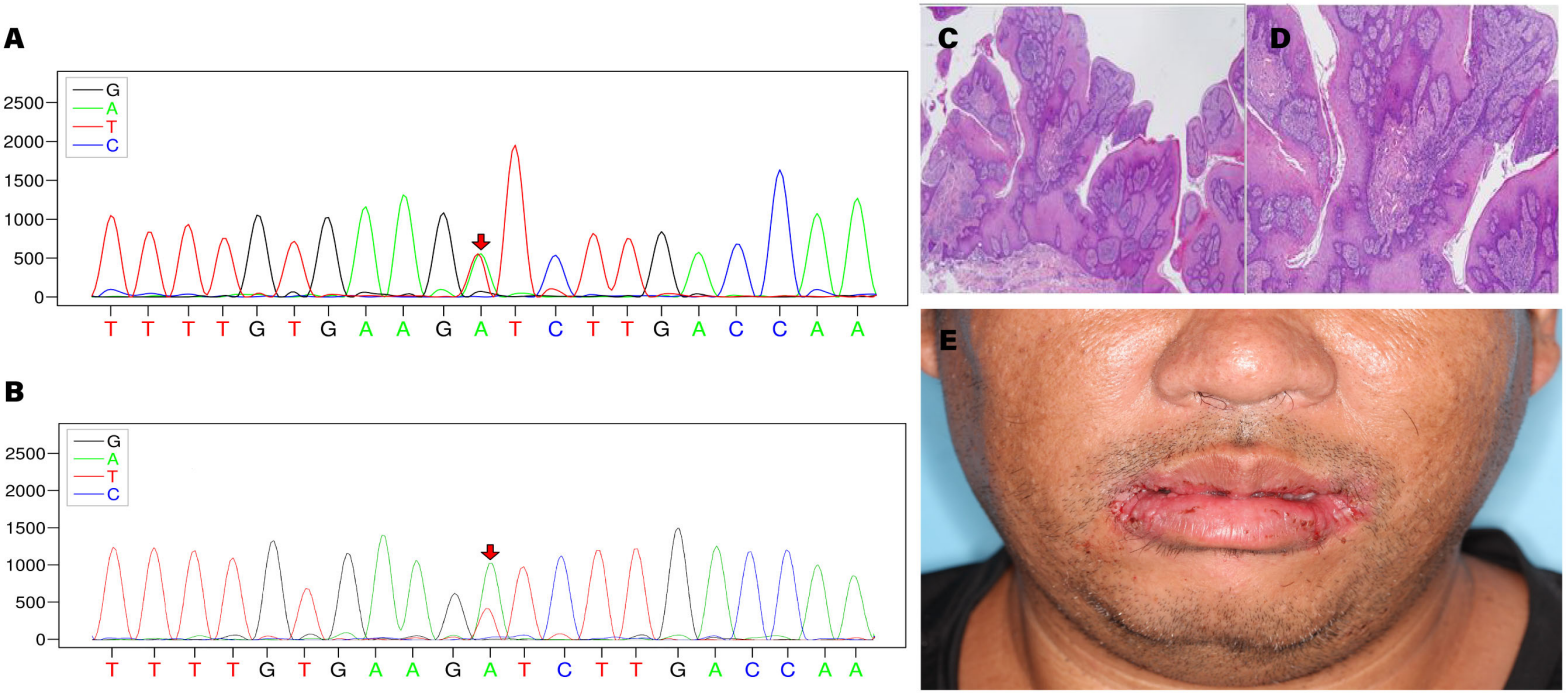
**(A)** Sequencing chromatogram of the proband. **(B)** Sequencing chromatogram of the proband’s younger brother. **(C, D)** Histopathological images of bilateral commissural lesions. **(E)** Follow-up examination of the proband at 2 weeks postoperatively.

Specific variants associated with the proband’s condition were identified, which are related to CS type 1 (OMIM:158350) and macrocephaly/autism syndrome (OMIM:605309). The proband exhibited the variant c.320A>T/p. Asp107Val in *PTEN* (NM_000314.8) at the chromosomal position chr10:89692836, indicating a missense mutation (**Figures 2A, B**). According to the ACMG guidelines, *PTEN*: NM_000314.8: c.320A>T (p. Asp107Val) is classified as pathogenic. The ACMG evidence supporting this classification includes PM2_supporting, PP3, PS4_supporting, PM6 (9), PS3_supporting (10), PP1. The REVEL score for the *PTEN* variant c.320A>T (chr10:89692836) is 0.862. The REVEL score prediction was performed using the REVEL Functional Predictions 2016-06-03 in the Golden Helix Genome Browse 3.0.0 software, as predicted by GHI. Additionally, it should be noted that the gnomAD database currently does not include this variant locus. In the proband, the gene mutation was found to be heterozygous. In the proband’s gene sequence, this mutation had a variant depth of 57, and it was observed 57 times out of 119 sequenced reads. Similarly, the proband’s younger brother was also found to be a carrier of the *PTEN* gene mutation, with a variant depth of 57 observed in 127 sequenced reads. The proband’s cousin did not have this gene mutation.

Based on the diagnostic criteria established by the International Cowden Consortium (11), a definitive diagnosis was made, and the proband subsequently underwent surgical intervention. Histopathological examination of the excised tissue revealed papillary hyperplasia of the stratified squamous epithelium with elongation of the rete ridges, displaying a grid-like pattern, along with mild focal abnormal proliferation in the bilateral commissures of the mouth. Additional observations included excessive keratinization with formation of keratin plugs in select regions, as well as micro abscesses on the surface of the local epithelium. Fibrous and vascular proliferation predominated in the subepithelial layer, accompanied by a notable presence of plasma cells, lymphocytes, and a minor population of neutrophils infiltrating the tissue. These findings were indicative of benign lesions (**Figures 2C, D**). The proband demonstrated a favorable prognosis following the surgical procedure (**Figure 2E**).

## 4 Discussion

We present a case of CS in a 41-year-old male proband, characterized by bilateral angle of mouth and lateral tongue lesions exhibiting an externally projecting symmetric growth pattern. The proband also displayed additional features such as macrocephaly, social interaction difficulties, and obesity. Similar oral lesion cases were identified within the proband’s family. Whole exome sequencing analysis revealed a *PTEN* gene mutation located at chr10:89692836. The transcript variant of this gene is NM_000314.8, and it harbors a c.320A>T variant resulting in an amino acid sequence change from aspartic acid (Asp) to valine (Val). This variant was observed in a heterozygous manner, with 57 out of 119 alleles carrying this mutation. Genetic testing of the proband’s younger brother confirmed the presence of the same gene mutation, while the cousin did not carry it.

CS, also known as multiple hamartoma syndrome, is an inherited disorder characterized by the presence of multiple hamartomas affecting various organs. These hamartomas originate from ectodermal, mesodermal, and endodermal tissues and have the potential for malignant transformation. The most prominent features of this syndrome include papillary mucosal overgrowth and papular skin lesions (12). Characteristic skin lesions manifest as flesh-colored verrucous papules with a diameter of 4mm, primarily distributed around the ears, sides of the neck, and especially in the periorbital, perinasal, and perioral regions. Small, translucent keratotic changes are observed on the palms and soles of the feet.

Originally, CS was perceived as primarily a dermatological condition until an increased risk for other manifestations was eventually identified (7, 13). This study observed the skin lesions of the proband (**Figures 1E, F**). The classical features of acral keratosis are well-supported in both early and contemporary research, with acral keratosis appearing on the palmoplantar surfaces and the dorsal hands and feet, manifesting as hyperkeratotic or palpable wart-like lesions (14–16). This aspect was corroborated in the proband (**Figure 1E**). Trichilemmomas are ectopic tumors of hair follicle tissue, considered the most prominent cutaneous manifestation in CS (8). In the initial case series, genetic testing was not yet available, but clinicians often observed the presence of multiple trichilemmomas on the face (17–19). Other published cases reported involvement in other areas such as the neck, axillae, and hands (20). Furthermore, there is research suggesting that CS may predispose patients to mucocutaneous HPV infection (6, 21). However, the proband in this study tested negative for HPV via serological examination. Given the difficulty in clinical diagnosis, the skin lesions in **Figures 1F, G** currently lack a definitive diagnosis; therefore, a mucosal biopsy should be obtained in clinically ambiguous lesions.

In many cases, patients also exhibit scrotal tongue and fissured tongue changes. Severe dental caries, macroglossia, gingival bleeding, high arched palate, hypoplastic uvula, and underdevelopment of the maxilla and mandible have been reported (22, 23). Visceral involvement in CS commonly includes thyroid adenomas, thyroid nodules, fibrocystic changes in the breasts, and colon polyps. These lesions often progress to malignancies such as thyroid cancer, breast cancer, and colon cancer, with a high rate of malignant transformation (24–26). Additionally, reports have indicated occurrences of malignant melanoma, liposarcoma, and acute myeloid leukemia. In addition, a few patients have been reported to present with multiple vascular malformations and lipomas (27). Other findings include macrocephaly, intellectual disability, ataxia, posterior spinal curvature (kyphoscoliosis), funnel chest (pectus excavatum), diverticulitis, hepatic, biliary, and pancreatic abnormalities, underdeveloped uterus, and pathological fractures (28). CS is an autosomal dominant hereditary disorder, with an equal prevalence in both males and females (29). Although CS is rare, it is of great importance to oral healthcare professionals because the majority of cases exhibit specific manifestations of this condition on the perioral skin and oral mucosa. This makes it easier for oral healthcare professionals to identify CS at an early stage compared to professionals in other specialties.

This case serves as a reminder to pay attention to the diverse manifestations of CS in oral clinical practice and to consider genetic testing for guiding diagnosis and individualized treatment. Although CS is primarily associated with *PTEN* gene mutations, recent research has also suggested an association between CS and neurodevelopmental disorders (30). Further studies are needed to gain a deeper understanding of the pathogenesis of CS and to enhance early diagnosis and management of this condition. Early detection enables effective prevention of the severe consequences associated with malignant transformations in multiple organs. To date, there is no effective treatment for CS, and palliative care is commonly employed (31, 32). In cases where the lesions impact function or result in ulceration and pain, local excision is more effective than cryotherapy. Once CS is suspected, a thorough systemic examination should be conducted to assess the presence of malignant manifestations.

## 5 Conclusion

In conclusion, this case holds significant importance in enhancing awareness of CS cases primarily presenting with oral clinical manifestations. Precise diagnosis and individualized treatment strategies will contribute to enhanced patient prognosis. However, further research is needed to gain a deeper understanding of the pathogenesis of CS and to strengthen early diagnosis and management of this condition.

## Data availability statement

The raw data supporting the conclusions of this article will be made available by the authors, without undue reservation.

## Ethics statement

The study received approval from the Ethics Committee of the Beijing Stomatological Hospital, Capital Medical University. Written informed consent was obtained from the individual(s) for the publication of any potentially identifiable images or data included in this article.

## Author contributions

WY: Conceptualization, Data curation, Formal analysis, Writing – original draft. YL: Methodology, Writing – original draft. HS: Investigation, Writing – review & editing. MS: Investigation, Writing – review & editing. LQ: Writing – original draft. XH: Project administration, Resources, Supervision, Writing – review & editing.

## References

[1] Clément D Frédéric C Bertrand D Djallel B Astrid DL Gérôme B . Impaired social cognition and fine dexterity in patients with Cowden syndrome associated with germline PTEN variants. J Med Genet (2023) 60(1):91–8. doi: 10.1136/jmedgenet-2021-107954 34937768

[2] Mester JL Tilot AK Rybicki LA Frazier TW 2nd Eng C . Analysis of prevalence and degree of macrocephaly in patients with germline PTEN mutations and of brain weight in Pten knock-in murine model. Eur J Hum Genet: EJHG (2011) 19(7):763–8. doi: 10.1038/ejhg.2011.20 PMC3137495 21343951

[3] Zhou XP Waite KA Pilarski R Hampel H Fernandez MJ Bos C . Germline PTEN promoter mutations and deletions in Cowden/Bannayan-Riley-Ruvalcaba syndrome result in aberrant PTEN protein and dysregulation of the phosphoinositol-3-kinase/Akt pathway. Am J Hum Genet (2003) 73(2):404–11. doi: 10.1086/377109 PMC1180378 12844284

[4] Tan MH Mester J Peterson C Yang Y Chen JL Rybicki LA . A clinical scoring system for selection of patients for PTEN mutation testing is proposed on the basis of a prospective study of 3042 probands. Am J Hum Genet (2011) 88(1):42–56. doi: 10.1016/j.ajhg.2010.11.013 21194675 PMC3014373

[5] Mester J Eng C . Estimate of *de novo* mutation frequency in probands with PTEN hamartoma tumor syndrome. Genet Med (2012) 14(9):819–22. doi: 10.1038/gim.2012.51 PMC3651836 22595938

[6] Hammerschmidt M Lourenco SV Nico MMS . A clinicopathological study of the oral lesions of Cowden disease. J Oral Pathol Med (2017) 46(8):637–43. doi: 10.1111/jop.12519 27889943

[7] Marshall M Otero D Niklander S Martinez-Flores R . Cowden’s syndrome diagnosed through oral lesions: A case report. J Clin Exp Dent (2021) 13(11):e1162–e6. doi: 10.4317/jced.58890 PMC8601691 34824704

[8] Flores IL Romo SA Tejeda Nava FJ Roger dos Santos Silva A Vargas PA Paes de Almeida O . Oral presentation of 10 patients with Cowden syndrome. Oral surgery Oral medicine Oral Pathol Oral radiology. (2014) 117(4):e301–10. doi: 10.1016/j.oooo.2014.01.015 24560406

[9] O’Roak BJ Stessman HA Boyle EA Witherspoon KT Martin B Lee C . Recurrent *de novo* mutations implicate novel genes underlying simplex autism risk. Nat Commun (2014) 5:5595. doi: 10.1038/ncomms6595 25418537 PMC4249945

[10] Brnich SE Abou Tayoun AN Couch FJ Cutting GR Greenblatt MS Heinen CD . Recommendations for application of the functional evidence PS3/BS3 criterion using the ACMG/AMP sequence variant interpretation framework. Genome Med (2019) 12(1):3. doi: 10.1186/s13073-019-0690-2 31892348 PMC6938631

[11] Eng C . Will the real Cowden syndrome please stand up: revised diagnostic criteria. J Med Genet (2000) 37(11):828–30. doi: 10.1136/jmg.37.11.828 PMC1734465 11073535

[12] Figueiredo L Leite PM Varela M Veiga F Fernandes A . A case report of a sclerotic fibroma of the oral mucosa. Cureus (2022) 14(8):e27627. doi: 10.7759/cureus.27627 36134098 PMC9481210

[13] Brownstein MH Wolf M Bikowski JB . Cowden’s disease: a cutaneous marker of breast cancer. Cancer (1978) 41(6):2393–8. doi: 10.1002/1097-0142(197806)41:6<2393::aid-cncr2820410644>3.0.co;2-k 657103

[14] Salem OS Steck WD . Cowden’s disease (multiple hamartoma and neoplasia syndrome). A case report and review of the English literature. J Am Acad Dermatol (1983) 8(5):686–96. doi: 10.1016/s0190-9622(83)70081-2 6863628

[15] Hanssen AM Fryns JP . Cowden syndrome. J Med Genet (1995) 32(2):117–9. doi: 10.1136/jmg.32.2.117 PMC1050232 7760320

[16] Oh JG Yoon CH Lee CW . Case of Cowden syndrome associated with eccrine angiomatous hamartoma. J Dermatol (2007) 34(2):135–7. doi: 10.1111/j.1346-8138.2006.00233.x 17239153

[17] Starink TM van der Veen JP Arwert F de Waal LP de Lange GG Gille JJ . The Cowden syndrome: a clinical and genetic study in 21 patients. Clin Genet (1986) 29(3):222–33. doi: 10.1111/j.1399-0004.1986.tb00816.x 3698331

[18] Carlson HE Burns TW Davenport SL Luger AM Spence MA Sparkes RS . Cowden disease: gene marker studies and measurements of epidermal growth factor. Am J Hum Genet (1986) 38(6):908–17. PMC1684853 3487976

[19] Starink TM Meijer CJ Brownstein MH . The cutaneous pathology of Cowden’s disease: new findings. J Cutaneous Pathol (1985) 12(2):83–93. doi: 10.1111/j.1600-0560.1985.tb01607.x 2582011

[20] Masmoudi A Chermi ZM Marrekchi S Raida BS Boudaya S Mseddi M . Cowden syndrome. J Dermatol Case Rep (2011) 5(1):8–13. doi: 10.3315/jdcr.2011.1063 21886759 PMC3163352

[21] Pilarski R Burt R Kohlman W Pho L Shannon KM Swisher E . Cowden syndrome and the PTEN hamartoma tumor syndrome: systematic review and revised diagnostic criteria. J Natl Cancer Inst (2013) 105(21):1607–16. doi: 10.1093/jnci/djt277 24136893

[22] Magaña M Landeta-Sa AP YL-F . Cowden disease: A review. Am J dermatopathology. (2022) 44(10):705–17. doi: 10.1097/dad.0000000000002234 36122333

[23] Sutera S Giachino DF Pelle A Zuntini R Pentenero M . Gingival overgrowths revealing PTEN hamartoma tumor syndrome: report of novel PTEN pathogenic variants. Biomedicines (2022) 11(1):81. doi: 10.3390/biomedicines11010081 PMC9855721 36672590

[24] Sokolova A Johnstone KJ McCart Reed AE Simpson PT Lakhani SR . Hereditary breast cancer: syndromes, tumour pathology and molecular testing. Histopathology (2023) 82(1):70–82. doi: 10.1111/his.14808 36468211 PMC10953374

[25] Rebuzzi F Ulivi P Tedaldi G . Genetic predisposition to colorectal cancer: how many and which genes to test? Int J Mol Sci (2023) 24(3):2137. doi: 10.3390/ijms24032137 PMC9916931 36768460

[26] Lee Y Oh YL . Thyroid pathology, a clue to PTEN hamartoma tumor syndrome. J Pathol Trans Med (2023) 57(3):178–83. doi: 10.4132/jptm.2023.03.04 PMC10209666 36977604

[27] Plana-Pla A Condal L Jaka A Blanco I Castellanos E Bielsa I . Verrucous epidermal nevus as a manifestation of a type 2 mosaic PTEN mutation in Cowden syndrome. Pediatr Dermatol (2023) 40(1):179–81. doi: 10.1111/pde.15116 PMC10087675 36151877

[28] Capitanio JF Mortini P . Brain and/or spinal cord tumors accompanied with other diseases or syndromes. Adv Exp Med Biol (2023) 1405:645–72. doi: 10.1007/978-3-031-23705-8_25 37452957

[29] Cavaillé M Crampon D Achim V Bubien V Uhrhammer N Privat M . Diagnosis of PTEN mosaicism: the relevance of additional tumor DNA sequencing. A case report and review of the literature. BMC Med Genomics (2023) 16(1):166. doi: 10.1186/s12920-023-01600-0 37442961 PMC10339495

[30] Hansen-Kiss E Beinkampen S Adler B Frazier T Prior T Erdman S . A retrospective chart review of the features of PTEN hamartoma tumour syndrome in children. J Med Genet (2017) 54(7):471–8. doi: 10.1136/jmedgenet-2016-104484 28526761

[31] Gülseren EŞ Ferda ÖH Şule Y Naz GL Aylin EG Şahin G . Sirolimus treatment of a PTEN hamartoma tumor syndrome presenting with melena. Turkish J Pediat (2022) 64(4):766–74. doi: 10.24953/turkjped.2021.5330 36082652

[32] Patini R Staderini E Gallenzi P . Multidisciplinary surgical management of Cowden syndrome: Report of a case. J Clin Exp Dent (2016) 8(4):e472–e4. doi: 10.4317/jced.52919 PMC5045699 27703620

